# An *Atoh1* CRE Knock-In Mouse Labels Motor Neurons Involved in Fine Motor Control

**DOI:** 10.1523/ENEURO.0221-20.2021

**Published:** 2021-01-21

**Authors:** Osita W. Ogujiofor, Iliodora V. Pop, Felipe Espinosa, Razaq O. Durodoye, Michael L. Viacheslavov, Rachel Jarvis, Mark A. Landy, Channabasavaiah B. Gurumurthy, Helen C. Lai

**Affiliations:** 1 Department of Neuroscience, University of Texas Southwestern Medical Center, Dallas, TX 75390; 2 Mouse Genome Engineering Core Facility, University of Nebraska Medical Center, Omaha, NE 68198; 3 Department of Pharmacology and Experimental Neuroscience, College of Medicine, University of Nebraska Medical Center, Omaha, NE 68198

**Keywords:** Atoh1, fine motor control, motor neurons, spinal cord

## Abstract

Motor neurons (MNs) innervating the digit muscles of the intrinsic hand (IH) and intrinsic foot (IF) control fine motor movements. The ability to reproducibly label specifically IH and IF MNs in mice would be a beneficial tool for studies focused on fine motor control. To this end, we find that a CRE knock-in mouse line of *Atoh1*, a developmentally expressed basic helix-loop-helix (bHLH) transcription factor, reliably expresses CRE-dependent reporter genes in ∼60% of the IH and IF MNs. We determine that CRE-dependent expression in IH and IF MNs is ectopic because an *Atoh1* mouse line driving FLPo recombinase does not label these MNs although other *Atoh1*-lineage neurons in the intermediate spinal cord are reliably identified. Furthermore, the CRE-dependent reporter expression is enriched in the IH and IF MN pools with much sparser labeling of other limb-innervating MN pools such as the tibialis anterior (TA), gastrocnemius (GS), quadricep (Q), and adductor (Ad). Lastly, we find that ectopic reporter expression begins postnatally and labels a mixture of α and γ-MNs. Altogether, the *Atoh1* CRE knock-in mouse strain might be a useful tool to explore the function and connectivity of MNs involved in fine motor control when combined with other genetic or viral strategies that can restrict labeling specifically to the IH and IF MNs. Accordingly, we provide an example of sparse labeling of IH and IF MNs using an intersectional genetic approach.

## Significance Statement

Motor neurons (MNs) of the intrinsic hand (IH) and intrinsic foot (IF) are reproducibly labeled in an ectopic manner postnatally using a CRE knock-in mouse line of the basic helix-loop-helix (bHLH) transcription factor *Atoh1*, serving as a useful genetic tool for future studies of fine motor control.

## Introduction

Motor neurons (MNs) innervating the muscles in the digits of the hand allow for exquisite control of fine motor movements required for dexterous skills, such as writing or sewing. Primates are known for their ability to precisely control individual digits of the hand, but skillful movements are not limited to the hand, as people born without arms are able to dexterously manipulate tools with their toes (Shubin et al., 1997; Sustaita et al., 2013; Dempsey-Jones et al., 2019). Precisely how MNs innervating the intrinsic hand (IH) and intrinsic foot (IF) muscles may differ in terms of function and connectivity compared with MNs that innervate limb muscles is unknown. Presumably, IH and IF MNs have unique properties and connectivity that contribute to their role in fine motor control, however, examination of the function of specifically IH and IF MNs is underexplored.

In part, lack of exploration of the IH and IF MNs is because of an inability to genetically distinguish these MNs from other limb MNs in mice. Mice also demonstrate remarkable manipulative dexterity when performing fine motor tasks such as eating dried pasta or grasping a pellet (Tennant et al., 2010; Yoshida and Isa, 2018) and, thus, represent a model organism to study fine motor skills. Developing a genetic tool that labels IH and IF MNs would have significant utility in interrogating the function and circuitry of fine motor control. To this end, we found that digit-innervating MNs were labeled using CRE-loxP recombination driven by the bHLH transcription factor atonal homolog 1, *Atoh1*, a transiently expressed gene in the dorsal-most part of the developing neural tube that is also known to specify excitatory (*Vglut2^+^*) spinal cord neurons that project rostrally to the hindbrain (Bermingham et al., 2001; Gowan et al., 2001; Sakai et al., 2012; Yuengert et al., 2015; Pop et al., 2020).

Here, we explore the features of the digit-innervating MNs labeled by *Atoh1* CRE-LoxP recombination. We find that labeling of IH and IF MNs is ectopic because an *Atoh1* mouse line expressing FLPo recombinase that we developed in the lab does not label IH and IF MNs, although it does label the expected *Atoh1*-lineage neurons in the intermediate spinal cord. The *Atoh1* CRE knock-in mouse labels a mixture of α-MNs and γ-MNs in the IH and IF MN pools postnatally, while sparsely labeling other limb-innervating MNs. We further endeavor to show the utility of the *Atoh1* CRE knock-in mouse using an intersectional genetic approach and discuss possible future methodologies to interrogate fine motor function and circuitry using this mouse strain.

## Materials and Methods

### Mouse strains

The following mouse strains were used: *Atoh1^Cre/+^* knock-in (Yang et al., 2010), *R26^LSL-tdTom/+^* (Ai14; Madisen et al., 2010), *R26^LSL-FSF-tdTom/+^* (Ai65; Madisen et al., 2015), *R26^FSF-tdTom/+^* was generated using CAG-Cre (Sakai and Miyazaki, 1997) crossed to Ai65, *R26^FSF-EGFP/+^* (named *RCE::RFrt* in Miyoshi et al., 2010), and *Chat^IRES-FLPo^* (Allaway et al., 2020). All mice were outbred and thus, are mixed strains (at least C57Bl/6J and ICR). *Atoh1^Cre/+^* knock-in mice crossed to reporter mice were screened for “dysregulated” expression as previously reported (Yuengert et al., 2015). All animal experiments were approved by the Institutional Animal Care and Use Committee at University of Texas Southwestern.

The *Atoh1^P2A-FLPo/+^* mouse was generated using the Easi-CRISPR approach (Quadros et al., 2017). Briefly, a long single stranded DNA cassette consisting of a viral peptide self-cleaving sequence [porcine teschovirus-1 2A (P2A); Kim et al., 2011] and the codon optimized flippase recombinase sequence (FLPo) were inserted after the last amino acid codon and before the stop codon of *Atoh1*. C57Bl/6N zygotes were microinjected with ribonucleoprotein complexes of Cas9 protein, tracrRNA, and crRNA (5′-TGACTCTGATGAGGCCAGTT-3′) along with a ssDNA megamer for homologous recombination (1497 bp containing 60 bp each 5′ and 3′ homology arms and the P2A-FLPo sequence; reagents were procured from IDT, microinjection service was provided by the UTSW Transgenic Mouse Core Facility). Assembling CRISPR reagents and microinjections were performed as previously described (Jacobi et al., 2017; Miura et al., 2018). The live born mice were first screened for insertion of the P2A-FLPo sequence and of those that were positive, one of the mice contained the full-length cassette. The cassette contained a minor mutation at the end of FLPo (the last isoleucine amino acid was changed to a serine), which could have occurred possibly because of an imprecise DNA repair event. Nevertheless, this amino acid change does not seem to affect the enzymatic function of FLPo. For genotyping, wild-type (WT) 321-bp and mutant 642-bp PCR products were detected using the following primers: WT forward 5′-CCCTAACAGCGATGATGGCACAGAAGG-3′, WT reverse 5′-GGGGATTGGAAGAGCTGCAGCCGTC-3′, and MUT reverse 5′-CGAACTGCAGCTGCAGGCTGGACACG-3′. Note that because the P2A sequence self-cleaves near its C terminus, 21 amino acids of the P2A sequence are fused to the C terminus of ATOH1.

### Tissue processing

Embryos were timed as embryonic day (E)0.5 on the day the vaginal plug was detected and P0 on the day of birth. Pregnant females were euthanized with CO_2_ and cervical dislocation, embryos dissected out of the uterus, and spinal cords dissected out. Embryonic spinal cords (E14.5) were fixed in 4% paraformaldehyde (PFA)/PBS for 2–3 h at 4°C. Early postnatal animals (postnatal day 7 (P7) or younger) were cooled on ice, decapitated, their spinal cords dissected out, and fixed in 4% PFA/PBS for 2 h at 4°C. Mice older than P14 were anesthetized with avertin (2,2,2-tribromoethanol; 0.025–0.030 ml of 0.04 m avertin in 2-methyl-2-butanol and distilled water/g mouse) and transcardially perfused, first with 0.012% w/v Heparin/PBS and then 4% PFA/PBS. A dorsal or ventral laminectomy exposed the spinal cord to the fixative. The spinal cords were fixed for 2 h and the brains overnight at 4°C. Tissue was washed in PBS for at least 1 d and cryoprotected in 30% sucrose dissolved in deionized water. Tissue was marked with 1% Alcian Blue in 3% acetic acid on one side to keep orientation and were then embedded in OCT (Tissue-Tek Optimal Cutting Temperature compound). Tissue was sectioned using a Leica CM1950 Cryostat.

### Immunohistochemistry and confocal imaging

Cryosections (30 μm) were blocked with PBS/1–3% normal goat or donkey serum/0.3% Triton X-100 (Sigma) for up to 1 h at room temperature (RT) and incubated overnight with primary antibody at 4°C. After washing three times with PBS, the appropriate secondary antibody (Alexa Fluor 488, 567, and/or 647, Invitrogen) was incubated for 1 h at RT. Sections were rinsed three times in PBS, mounted with Aquapolymount (Polysciences Inc.), and coverslipped (Fisher). The following primary antibodies and dilutions were used: 1:500 rabbit anti-dsRed (Clontech), 1:100 goat anti-choline acetyltransferase (ChAT; Millipore Sigma), 1:1000 rabbit anti-matrix metalloproteinase-9 (MMP9; Abcam), 1:8000 rabbit anti-HB9 (gift of Sam Pfaff, Salk Institute), 1:3000 guinea pig anti-copine-4 (CPNE4) and 1:8000 guinea pig anti-fidgetin (FIGN; gifts of Tom Jessell, Columbia University), 1:500 mouse anti-NEUN (Millipore Sigma), 1:100 mouse anti-ERR3 (R&D Systems), 1:3000 α-bungarotoxin (BTX) 488 (Invitrogen), 1:1000 rabbit anti-Syntaxin1 (gift of Thomas Südhof, Stanford University), and 1:1000 guinea pig anti-vesicular glutamate transporter 1 (VGLUT1; Millipore Sigma). Sections were referenced to the Christopher Reeves Spinal Cord Atlas (Watson et al., 2009).

Fluorescent images were taken on a Zeiss LSM710 or LSM880 confocal microscope with an appropriate optical slice (0.5–10 μm) depending on the image. Images were pseudocolored using a magenta/green/blue or magenta/yellow/cyan color scheme using Adobe Photoshop (Adobe) or Fiji (Schindelin et al., 2012).

### CTB muscle injections

Mice aged P14 were anesthetized using isoflurane and prepared for injections into muscle. An approximate total of 500–750 nl of cholera toxin subunit B (CTB) Alexa Fluor 488 or 647 conjugate (Invitrogen; Nanoject II, Drummond Scientific) was injected into two to three different locations in the left forepaw (IH MN pool) or hindpaw (IF MN pool) in 50.6 nl increments. For injections into the tibialis anterior (TA), gastrocnemius (GS), quadricep (Q), or adductor (Ad), the area of the skin above the muscle was shaved and 70% ethanol and betadine (Avrio Health L.P.) applied. An incision was made above the muscle and 500–750 nl of the CTB-conjugated Alexa Fluor was injected into three to four different locations directly into the muscle. The incision was closed with surgical glue (Henry Schein Medical). Carprofen (5 mg/kg) was administered daily 3 d after surgery. Spinal cords were harvested 5 d after injection. For injections at P0, mice were anesthetized with isoflurane and injected with <250 nl CTB-488 or 647 in one or two different locations of the forepaw and hindpaw and harvested 3 d later.

### Experimental design and statistical tests

All details for number of sections counted, biological replicates, and male and female tissue analyzed are given in Results. No statistical tests were required as quantitation of the percentage of particular markers in any given MN pool were not directly compared with each other. Mean ± SEM are reported throughout. For samples with *n* = 2, the SEM is equal to half of the range between the two data points.

## Results

### *Atoh1^Cre/+^* knock-in mice label MN pools involved in fine motor control

**Figure 1.**
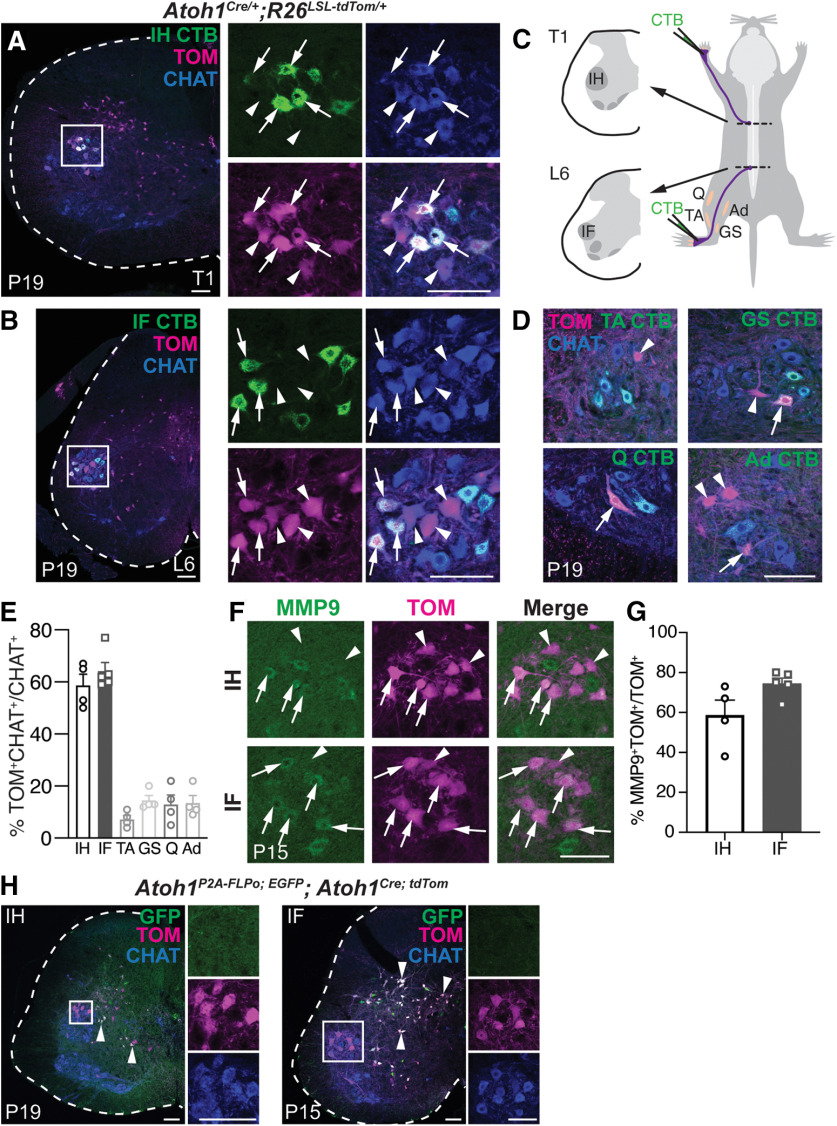
The *Atoh1^Cre/+^* knock-in mouse line labels the IH and IF MN pools. ***A***, ***B***, Injection of the retrograde tracer CTB-488 into the forepaw and hindpaw labels the IH and IF MN pools, which are labeled with tdTomato (TOM) in *Atoh1^Cre/+^* knock-in mice. MN pools are identified with ChAT antibody. Arrows, CTB^+^TOM^+^ChAT^+^; arrowheads, CTB^–^TOM^+^ChAT^+^. ***C***, Diagram of CTB-488 injections into the IH and IF MN pools, which are located at T1 and L6. ***D***, Injection of CTB-488 into the TA, GS, Q, and Ad muscles retrogradely labels those MN pools, which are sparsely labeled with TOM in *Atoh1^Cre/+^* knock-in mice. Arrows, CTB^+^TOM^+^ChAT^+^; arrowheads, CTB^–^TOM^+^ChAT^+^. ***E***, Percentage of the IH, IF, TA, GS, Q, and Ad MN pools that are labeled TOM^+^ in *Atoh1^Cre/+^* knock-in mice. ***F***, ***G***, Some of the TOM^+^ IH MNs and IF MNs are fast twitch MNs (MMP9^+^). Arrows, MMP9^+^TOM^+^; arrowheads, MMP9^–^TOM^+^. ***H***, *Atoh1*-lineage neurons in the intermediate spinal cord have extensive overlap in *Atoh1^P2A-FLPo;^*^EGFP^ mice crossed to *Atoh1^Cre; tdTom^* mice (arrowheads, GFP^+^TOM^+^, white). However, IH and IF MNs are only labeled in the *Atoh1^Cre/+^* mice (TOM^+^) and not in the *Atoh1^P2A-FLPo/+^* mice (GFP^–^; inset). P, postnatal; T, thoracic; L, lumbar. Scale bars: 100 μm.

We observed using CRE-lineage tracing strategies (*Atoh1^Cre/+^* knock-in mice crossed to tdTomato (TOM) reporter mice (*R26^LSL-Tom^*, Ai14)) (Yang et al., 2010; Madisen et al., 2010) that subsets of MNs expressing ChAT were labeled in the spinal cord (Fig. 1*A*,*B*, arrows and arrowheads). Based on the anatomic location of the MN pools along the rostral-caudal axis, we predicted that the *Atoh1^Cre/+^* line labeled MNs of the IH and IF in thoracic 1 (T1) and lumbar 6 (L6) areas of the spinal cord (Fig. 1*C*; Watson et al., 2009). We injected the forepaw and hindpaw with the retrograde tracer CTB conjugated to Alexa Fluor 488 (CTB-488) and verified that the IH and IF MN pools were *Atoh1^Cre/+^* TOM^+^ MNs (Fig. 1*A*,*B*, arrows). Note that the TOM^+^ cell bodies in the intermediate spinal cord are from other *Atoh1*-lineage interneurons involved in the proprioceptive system (Yuengert et al., 2015; Pop et al., 2020).

**Figure 2.**
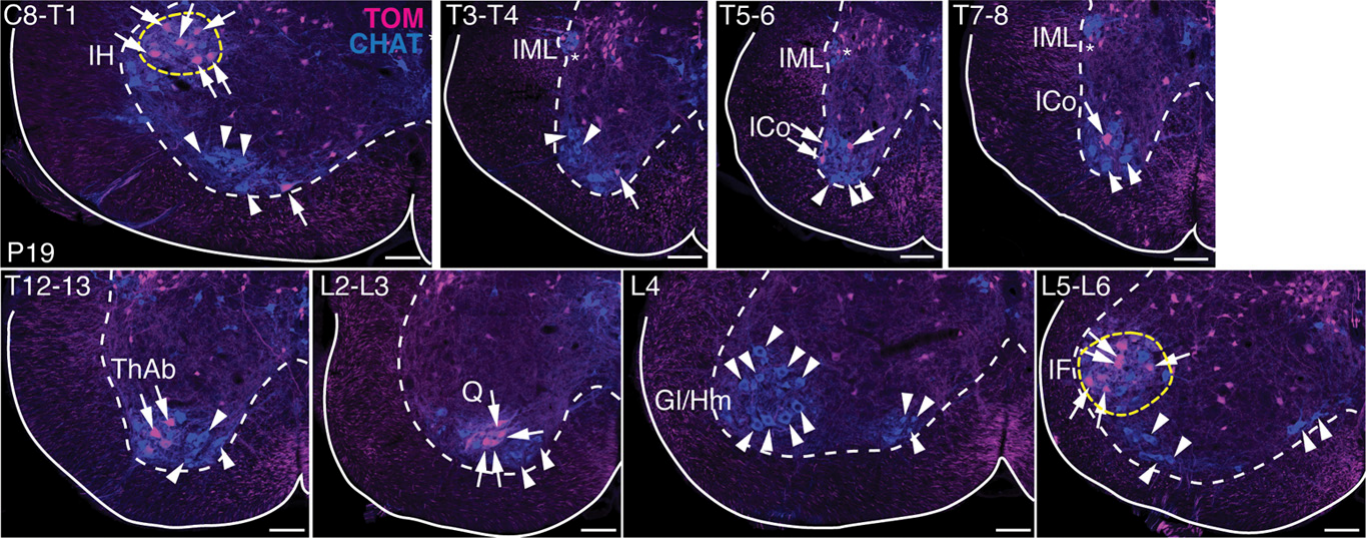
Distribution of MNs labeled in the *Atoh1^Cre/+^* knock-in mouse throughout the spinal cord. Representative images throughout the rostral-caudal axis of *Atoh1^Cre/+^* knock-in mice crossed to the TOM reporter mouse show that TOM labels MNs mainly in the IH and IF MN pools (yellow dashed lines) with sparser labeling of MNs in other MN pools (arrows). Some MN pools have no TOM^+^ expression (arrowheads). TOM^+^ChAT^+^ cells in the IML are sympathetic pre-MNs (asterisks). IML, intermediolateral nucleus; ICo, intercostal; ThAb, thoracic abductor; Q, quadricep; Gl, gluteus; Hm, Hamstring. Scale bars: 100 μm.

To see whether the labeling of *Atoh1^Cre/+^* TOM^+^ MNs was specific to the IH and IF MN pools, we injected CTB-488 into the TA, GS, Q, and Ad muscles and found that those MN pools had much fewer TOM^+^ MNs (Fig. 1*D*, arrows and arrowheads). In addition, we sampled sections throughout the rostral-caudal axis of the spinal cord in *Atoh1^Cre/+^* mice and found that few other MN pools had TOM^+^ expression (Fig. 2, arrows). Altogether, the TOM^+^ MNs labeled ∼60% of the IH and IF MN pools at P19 [IH: 59 ± 4%, *n* = 4, 1:3 male (M): female (F), four to eight half sections/*n*; IF: 64 ± 3%, *n* = 5, 1:4 M:F, two to eight half sections/*n*; TA: 7 ± 2%, *n* = 4, 2:2 M:F, two half sections/*n*; GS: 14 ± 2%, *n* = 4, 2:2 M:F, two to four half sections/*n*; Q: 13 ± 4%, *n* = 4, 2:2 M:F, one to three half sections/*n*; Ad: 13 ± 3%, *n* = 4, 2:2 M:F, one to six half sections/*n*. MN areas located by CTB-488 injection into appropriate muscle group; Fig. 1*E*]. We estimate that the total number of ChAT^+^ MNs in the IH and IF MN pools at P19 on one side is 410 ± 72 neurons for the IH MN pool (*n* = 3, 1:2 M:F, three to four half sections/*n*) and 337 ± 7 SEM neurons for the IF MN pool (*n* = 3, 0:3 M:F, three half sections/*n*). Counts of sections represented a tenth of the MN pool, so the estimated total number of ChAT^+^ neurons were the final counts multiplied by ten. Because the TOM^+^ MNs comprised ∼60% of the IH and IF MN pools, we hypothesized that the *Atoh1^Cre/+^* mice might be labeling a particular functional class of MNs such as fast or slow twitch MNs. However, we found that only a subset of the IH and IF TOM^+^ MNs were labeled by the marker for fast MNs, MMP9, indicating that the IH and IF TOM^+^ MNs are a mixture of fast and slow MNs (IH: 59 ± 8%, *n* = 4, 3:1 M:F, three half sections/*n*; IF: 74 ± 3%, *n* = 5, 3:2 M:F, three half sections/*n*; Fig. 1*F*,*G*, arrows; Kaplan et al., 2014).

Developmentally, all cholinergic MNs derive from a progenitor domain expressing the basic helix-loop-helix (bHLH) oligodendrocyte transcription factor 2 (*Olig2*) in the ventral neural tube (Lu et al., 2002), not the transiently-expressed *Atoh1* dorsal-most progenitor domain (Lai et al., 2016). Therefore, we tested whether *Atoh1* was expressed in MNs by *in situ* hybridization (Gowan et al., 2001 for ISH probe) and RNAscope of *Atoh1* at P14–P15 and P22, ages that we knew had TOM^+^ expression, but we were unable to detect any signal at the mRNA level (our unpublished observations). Therefore, to corroborate the labeling of IH and IF TOM^+^ MNs in the *Atoh1^Cre/+^* mouse line, we crossed these mice to an *Atoh1^P2A-FLPo/+^* mouse and FLPo-dependent GFP reporter mouse (*R26^FSF-EGFP/+^*) such that neurons from the *Atoh1^Cre/+^* mouse line were labeled with tdTomato and those from the *Atoh1^P2A-FLPo/+^* mouse were labeled with EGFP. Strikingly, while the IH and IF MN pools were TOM^+^, they were clearly GFP^–^ (Fig. 1*H*, insets) indicating that the TOM^+^ IH and IF MNs are ectopically labeled in the *Atoh1^Cre/+^* mice, either because of differences in CRE or FLPo recombinase expression themselves or differences in recombination efficiency in the CRE and FLPo lines. Notably, *Atoh1*-lineage neurons in the intermediate spinal cord had substantial overlap of GFP^+^ and TOM^+^ fluorescence (Fig. 1*H*, arrowheads) indicating these neurons are reliably from the *Atoh1*-lineage.

### *Atoh1^Cre/+^* knock-in mice label IH and IF MN pools postnatally

**Figure 3.**
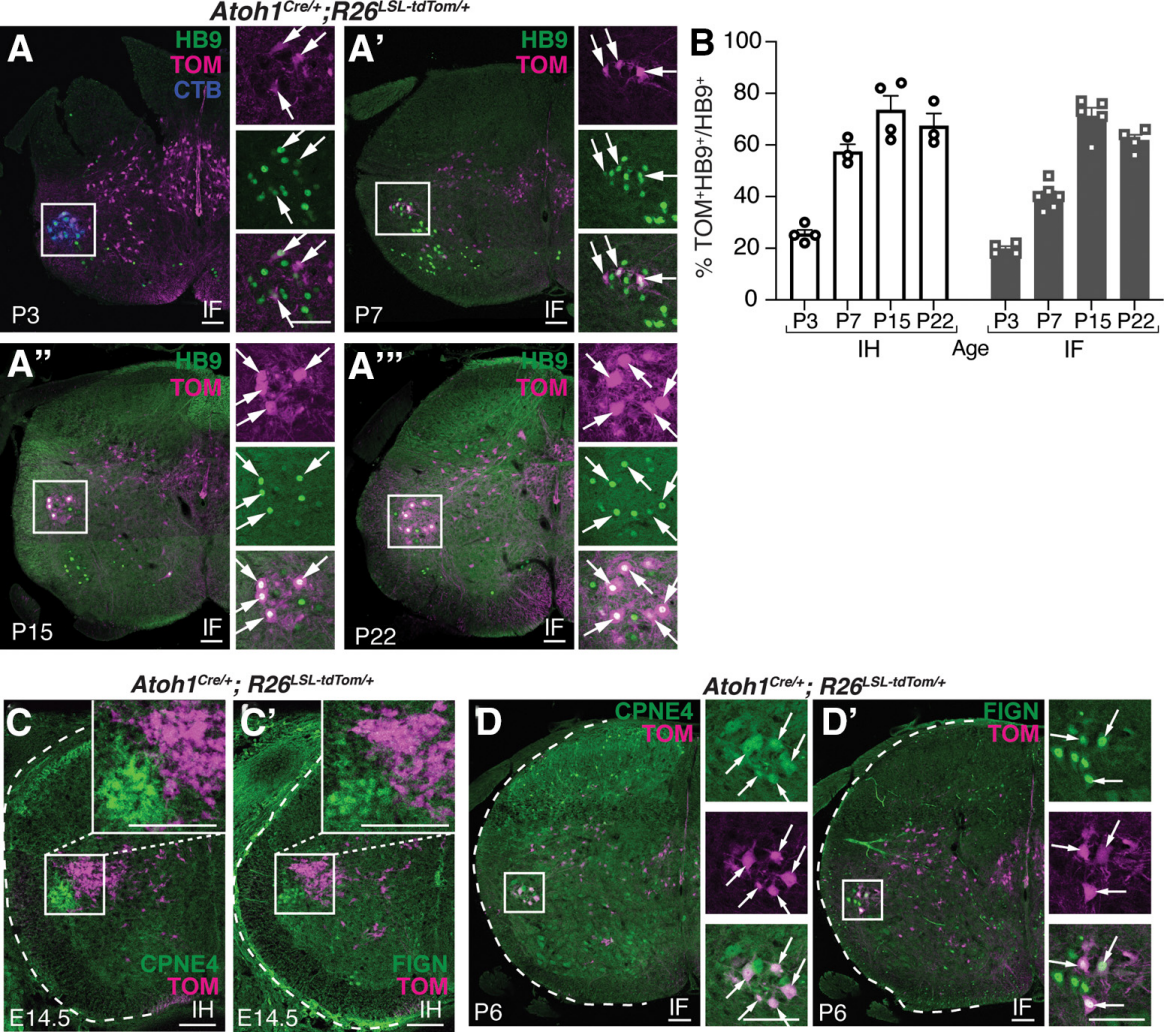
The *Atoh1^Cre/+^* knock-in mouse labels IH and IF MN pools postnatally. ***A–A’’’***, TOM^+^ labeling of the IF MN pool at several postnatal time points. TOM^+^HB9^+^ neurons, arrows. In ***A***, CTB (blue) was injected into the hindpaw to identify the IF MN pool. In ***A’–A’’’***, the IF MN pool was identified by location in the lumbar spinal cord. ***B***, Percentage of TOM^+^ neurons in the IH or IF MN pools at several postnatal time points. The IH and IF MN pools were identified by CTB injection into the forepaw and hindpaw at P0 and tissue harvested 3 d later for the P3 time point. IH and IF MN pools for P7, P15, and P22 were identified by location in the lumbar spinal cord. ***C–C’***, At E14.5, TOM^+^ neurons are CPNE4^–^ and FIGN^–^. ***D–D’***, At P6, TOM^+^ neurons are CPNE4^+^ and FIGN^+^ (arrows). Scale bars: 100 μm.

Because the *Atoh1^Cre/+^* line was labeling IH and IF MNs ectopically, we sought to determine when the ectopic expression occurred during development. We found that at E14.5 when the IH and IF MNs first start expressing the unique markers CPNE4 and FIGN (Mendelsohn et al., 2017), the IH and IF MN pools were not yet TOM^+^ (Fig. 3*C*,*C’*). In contrast, at postnatal time points, we found that TOM^+^ expression started around P3 and gradually increased until it reached ∼60% in the IH and IF MN pools at P22 using the homeobox transcription factor, HB9 as a marker for MN pools (Arber et al., 1999; P3: IH, 26 ± 2%, *n* = 4, sex was not noted because of young age, four to six half sections/*n*; IF, 20 ± 1%, *n* = 4, sex was not noted because of young age, five to six half sections/*n*; P7: IH, 57 ± 3%, *n* = 3, 1:2 M:F, two to six half sections/*n*; IF, 40 ± 2%, *n* = 6, 2:4 M:F, three to four half sections/*n*; P15: IH, 74 ± 6%, *n* = 4, 3:1 M:F, four half sections/*n*; IF, 71 ± 3%, *n* = 5, 3:2 M:F, four half sections/*n*; P22: IH, 67 ± 5%, *n* = 3, 2:1 M:F, four to eight half sections/*n*; IF, 62 ± 2%, *n* = 4, 1:3 M:F, four to eight half sections/*n*; Fig. 3*A–A’’’*,*B*). In addition, we confirmed that the IF TOM^+^ MNs colocalized with the specific markers CPNE4 and FIGN postnatally (Fig. 3*D*,*D’*).

### IH and IF MN pools labeled with *Atoh1^Cre/+^* knock-in mice are both α and γ-MNs

**Figure 4.**
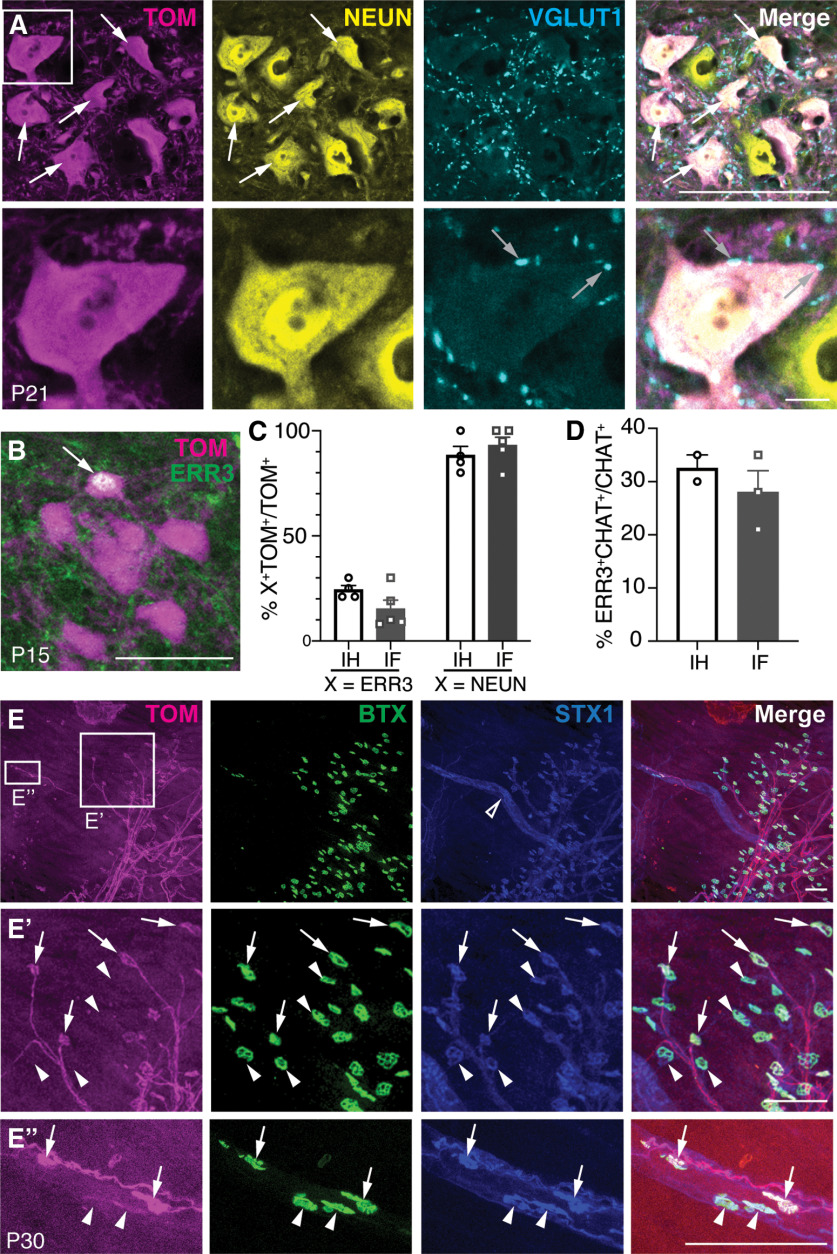
Both α-MNs and γ-MNs are labeled in the *Atoh1^Cre/+^* knock-in mouse. ***A***, TOM^+^ MNs in the IF MN pool are NEUN^+^ (arrows) and have closely apposed VGLUT1^+^ boutons (gray arrows). ***B***, Some TOM^+^ IF MNs are also ERR3^+^ (arrow). ***C***, Percentage of the TOM^+^ MNs in the IH and IF that are ERR3^+^ (γ-MN marker) or NEUN^+^ (α-MN marker). ***D***, Percentage of the IH and IF MN pools that are ERR3^+^. ***E–E’’***, TOM^+^ axons in the hindpaw lumbrical muscle show the NMJ innervating extrafusal muscle (***E’***, arrows, BTX^+^STX1^+^TOM^+^). TOM^+^ axons also innervate the intrafusal muscle spindle (***E***, open arrowhead; ***E’’***, arrows, BTX^+^STX1^+^TOM^+^). Arrowheads indicate motor endplates that are TOM^-^. Scale bars: 100 μm; 10 μm (***A***, inset).

Because *Atoh1^Cre/+^*TOM^+^ MNs are enriched in only a subset (∼60%) of IH and IF MNs, we asked whether they were demarcating a specific type of MN (α or γ). α-MNs innervate the striated extrafusal muscle, are marked by the neuronal marker, NEUN, and receive VGLUT1^+^ proprioceptive inputs (Friese et al., 2009; Manuel and Zytnicki, 2011; Ashrafi et al., 2012). γ-MNs innervate the intrafusal muscle spindles and express estrogen-related receptor γ (ERR3^+^; Friese et al., 2009). Immunostaining for α-MN and γ-MN markers, we found that *Atoh1^Cre/+^*TOM^+^ MNs were a mixture of both α-MNs and γ-MNs (% ERR3^+^: IH, 24 ± 2%, *n* = 4, 3:1 M:F, three to four half sections/*n*, IF, 15 ± 4%, *n* = 5, 3:2 M:F, two to four half sections/*n*; % NEUN^+^: IH, 88 ± 4%, *n* = 4, 3:1 M:F, three half sections/*n*, IF, 93 ± 4%, *n* = 5, 3:2 M:F, three half sections/*n*; Fig. 4*A–C*). Counts for the percentage of TOM^+^ neurons that are ERR3^+^ or NEUN^+^ were performed on different sets of sections because of the fact that they are both mouse antibodies. Therefore, the percentages do not necessarily add up to 100% and the error in counts may be attributed to variability between sections. The NEUN^+^ TOM^+^ MNs also received VGLUT1^+^ proprioceptive inputs (Fig. 4*A*). We confirmed that ∼30% of MNs are γ-MNs in the IH and IF MN pool as has been reported for other MN pools (IH: 33 ± 3%, *n* = 2, 2:0 M:F, three half sections/*n*, IF: 28 ± 4%, *n* = 3, 2:1 M:F, two to three half sections/*n*; Fig. 4*D*; Friese et al., 2009). Therefore, our results suggest that the *Atoh1^Cre/+^*mouse line has a slight preference for labeling α-MNs. Furthermore, imaging of the hindpaw lumbrical muscle found TOM^+^ axons innervating both the extrafusal (Fig. 4*E’*, arrows) and intrafusal muscle (Fig. 4*E’’*, arrows). BTX^+^ identifies the neuromuscular junctions (NMJs) and syntaxin (STX1^+^) identifies the muscle spindle (Fig. 4*E*, open arrowhead) and NMJs. Note that not all NMJs are TOM^+^ (Fig. 4*E’–E’’*, arrowheads) consistent with the fact that only ∼60% of the IH and IF MN pools are TOM^+^.

### Intersectional strategy for labeling IH and IF MNs

**Figure 5.**
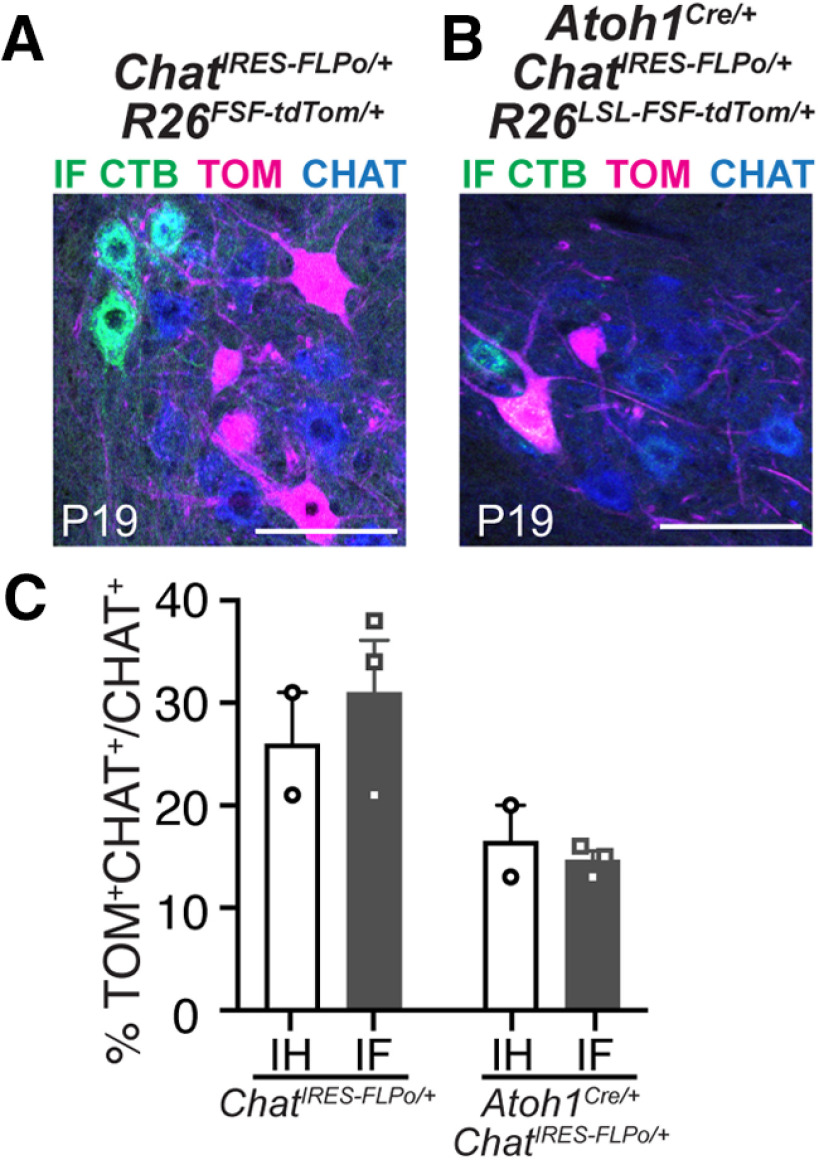
Labeling of IH and IF MNs using an intersectional genetic approach. ***A***, *Chat^IRES-FLPo/+^* mice crossed to a FLPo-dependent tdTomato reporter labels the IF MN pool, identified by injection of CTB into the hindpaw. ChAT Ab identifies the entire MN pool. ***B***, The IF MN pool is sparsely labeled using an intersectional cross (*Atoh^Cre/+^*; *Chat^IRES-FLPo/+^*). ***C***, Percentage of the IH and IF MN pools labeled in *Chat^IRES-FLPo/+^* mice and *Atoh^Cre/+^*; *Chat^IRES-FLPo/+^* mice. IH and IF MN pools were identified by retrograde labeling with CTB. Scale bars: 100 μm.

Because *Atoh1*-lineage neurons are glutamatergic and reside in the intermediate spinal cord, we sought a way to isolate specifically the ectopically labeled IH and IF MNs using an intersectional strategy crossing *Atoh1^Cre/+^*and *Chat^IRES-FLPo/+^* alleles to the intersectional tdTomato reporter (*R26^LSL-FSF-tdTomato^*, Ai65). We found that labeling of the IH and IF MNs was sparse in the *Chat^IRES-FLPo/+^* mouse line alone with only ∼30% of the MN pool being labeled (IH, 26 ± 5%, *n* = 2, 2:0 M:F, six half sections/*n*; IF, 31 ± 5%, *n* = 3, 3:0 M:F, 4–10 half sections/*n*; Fig. 5*A*,*C*). Thus, for the intersection of *Atoh^Cre/+^* and *Chat^IRES-FLPo/+^*, very few IH and IF MNs were labeled (IH, 17 ± 4%, *n* = 2, 1:1 M:F, two to three sections/*n*; IF, 15 ± 1%, *n* = 3, 2:1 M:F, three to four half sections/*n*; Fig. 5*B*,*C*). Therefore, this intersectional cross could be useful for sparse labeling of the IH and IF MN pools.

## Discussion

To understand how dexterous movements of the hand and foot is achieved, we must have some knowledge about the function and circuitry of MNs involved in fine motor control. Thus, obtaining genetic tools in mice that could reproducibly label the IH and IF MNs could help address questions as to how these neurons differ perhaps in their electrophysiological properties and connectivity compared with MNs innervating limb muscles involved in gross motor control. To this end, we found that MNs that innervate the IH and IF are labeled in *Atoh1^Cre/+^*mice. We found that labeling of the IH and IF MNs is ectopic and occurs postnatally resulting in ∼60% of IH and IF MNs being labeled by approximately three weeks of age with a slight preference for labeling α-MNs over γ-MNs. Here, we discuss some possible uses for the *Atoh1^Cre/+^*mouse line for future studies interrogating fine motor control circuits.

If IH and IF MNs could be isolated specifically without labeling other neurons in the nervous system, then a number of genetic tools could be used to either manipulate the activity of these MNs (i.e., optogenetic and/or chemogenetic approaches) or identify the inputs to these MNs (i.e., transsynaptic rabies virus tracing). One approach to isolate IH and IF MNs would be to use an intersectional genetic strategy crossing the *Atoh1^Cre/+^*mice to mice expressing FLPo recombinase in the IH and IF MNs, but not in other overlapping *Atoh1^Cre/+^*domains. We attempted an initial intersectional cross of *Atoh1^Cre/+^*to *Chat^IRES-FLPo/+^* and found sparse labeling of the IH and IF MNs because of inefficient recombination in these MNs by the *Chat^IRES-FLPo/+^* line. While this particular intersectional cross could be useful for sparse labeling of the IH and IF MNs for anatomic studies, one must keep in mind that *Atoh1^Cre/+^*and *Chat^IRES-FLPo/+^* intersect in other areas of the central nervous system such as occasionally in other MN pools (Fig. 2) and in *Atoh1*-lineage cholinergic neurons in the pedunculopontine tegmentum (PPTg) and the lateral dorsal tegmentum (LDTg) (our unpublished observations; Rose et al., 2009). Thus, an alternate cross is required to separate out the IH and IF MNs.

Moving forward, we propose that other FLPo-recombinase lines could be used in conjunction with the *Atoh1^Cre/+^* mouse line to isolate the IH and IF MNs. For example, FLPo driven by *Hb9*, *Fign*, or *Cpne4*, which we have shown colocalize with the IH and IF MNs labeled in the *Atoh1^Cre/+^* line, but not in other *Atoh1*-lineage neurons in the intermediate spinal cord (Fig. 3), would be promising candidates. *Hb9*, however, has the same caveat as *Chat* in that other MN pools throughout the spinal cord may intersect with *Atoh1^Cre/+^* expression. IH and IF MNs were found to contain a repertoire of unique molecular markers compared with neighboring limb-innervating MNs suggesting a unique developmental program (Mendelsohn et al., 2017). Indeed, the transcriptional landscape in IH and IF MNs appears to preferentially activate the CRE recombinase in *Atoh1^Cre/+^* mice. Of these unique molecular markers, other candidate genes include *Ecrg4*, *Reg3b*, *Serpinf1*, and *Pirt* (Mendelsohn et al., 2017), although these would need further characterization of spatial and temporal overlap with the *Atoh1^Cre/+^* line to determine their suitability for future studies. Furthermore, *Osmr* and *Col8a1* could be considered for exploration of specifically IF MNs (Mendelsohn et al., 2017).

An alternate strategy would be to use viruses to restrict labeling to just the IH or IF MN pools. For example, the *Atoh1^Cre/+^* line could be crossed to an intersectional line expressing a reporter of choice (i.e., fluorescent, optogenetic, or chemogenetic reporter) and an AAV-FLPo injected into either the spinal cord or in the forepaw or hindpaw to be taken up retrogradely to the IH and IF MNs. However, a caveat of this approach is the potential damage to the spinal cord or muscles of the forepaw and hindpaw because of the needle injection. Thus, appropriate injection controls would be needed with this approach.

Altogether, we present here that the *Atoh1^Cre/+^* mouse consistently labels MNs of the IH and IF and that the *Atoh1^Cre/+^* mouse could be used to probe the function and connectivity of MNs in fine motor control. Our findings also serve as a cautionary tale of relying on CRE-recombinase mouse lines to faithfully report endogenous gene expression and speak to the need for careful follow-up experiments to appropriately interpret reporter expression.

## Synthesis

Reviewing Editor: Lynda Erskine, University of Aberdeen Decisions are customarily a result of the Reviewing Editor and the peer reviewers coming together and discussing their recommendations until a consensus is reached. When revisions are invited, a fact-based synthesis statement explaining their decision and outlining what is needed to prepare a revision will be listed below. The following reviewer(s) agreed to reveal their identity: David Ladle. Note: If this manuscript was transferred from JNeurosci and a decision was made to accept the manuscript without peer review, a brief statement to this effect will instead be what is listed below.

Your manuscript has been reviewed by myself and 2 additional reviewers. We are all agree that the manuscript comprises an extremely detailed set of analyses and that the images are beautiful and well presented. Overall, we felt that the characterisation of this animal model is potentially valuable for the field. However, we have a number of concerns about the framing of the manuscript and some of the conclusions drawn. In particular, we felt that the manuscript would benefit from extensive re-writing to focus on the characterisation of the IH/IF motor neurons labelled in the Atoh1cre mouse, a toning down or removal of the claims made regarding the developmental programmes used by these cells, more consideration that the cre-mediated recombination driven in the IH/IF motor neurons may be due to ectopic rather than endogenous Atoh1 expression in these cells, and a more frank appraisal of the potential usefulness of the Atoh1cre line for future functional experiments.

Below is a list of comments that, following consultation between the reviewers and myself, we are all agreed are essential to address in a revised manuscript.

1. A potential limitation of the Atoh1cre line for use in future functional experiments is that this line does not drive recombination specifically in IH/IF motor neurons. This is exemplified by the fact that the authors were unable to use this mouse line in their experiments aimed at tracing collaterals to the cerebellum (due to recombination being driven in other neuronal cell types). In their revised manuscript, the authors need to discuss in more detail these limitations and be realistic about the potential usefulness of this tool for studying IH/IF motor neurons.

The impact of the paper would be increased substantially by demonstrating the usefulness of this Cre line (e.g. to isolate and profile IH/IF motor neurons or show a role for Atoh1 in these motor neurons). However, we appreciate that such experiments may be very time consuming and beyond the scope of this manuscript.

2. The focus and overall “story” of the paper is not clear. The 3 main findings are not entirely connected to each other (more on this below). Moreover, the authors set up questions that they do not actually address. For example, the authors set up at the start of the introduction the question of how digit innervating motor neurons adopt their unique identity. However, they do not address this question in the manuscript. What they actually do is characterise the features of the digit innervating motor neurons labelled by Atoh1cre. The manuscript would benefit from re-writing to make the focus, take home message and contribution to the field clearer.

3. The authors’ claim that the labelling of IH /IF motor neurons by Atoh1cre mediated recombination supports that these motor neurons have a unique developmental history. However, the evidence supporting this claim is not compelling and more work would be required to substantiate this (for example, demonstrating a role for Atoh1 in their specification). If the labelling of these cells is because of ectopic expression in the Atoh1cre line (which appears entirely plausible due to the inability to detect endogenous Atoh1 expression in IH/IF motor neurons), what is this actually telling us about the developmental history of these cells? Moreover, because the authors only demonstrate that the Cre-line labels IH/IF neurons, but do not demonstrate a functional role for Atoh1 during development of these cells, their findings do not provide any insights into how digit innervating MNs adopt their unique identities.

In figure 2, one could argue that TOM+ MN labeling is enriched in a ventral population of L2-L3 MNs. From the reference cartoon above the image it is difficult to determine if these TOM+ MNs belong to the psoas or quadricep (possibly rectus femoris) pools. Rectus femoris MNs are most definitely limb MNs, but have a genetic identity distinct from other quadriceps MNs, in that they transiently express Pea3 (Etv4) and are negative for Er81 (Etv1) (De Marco Garcia & Jessell 2008). This observation would also seem to run counter to the authors’ conclusion that IF/IH MNs have a developmental history distinct from other limb MNs.

The authors’ claim that their findings provide information on the developmental histories of IH/IF motor neurons therefore needs to be toned down substantially or removed.

4. The authors tried to test if IH/IF motor neurons send collaterals to the cerebellum. Multiple approaches were used, all with caveats. Additionally, it appears that only a small number of animals were analysed in some experiments, limiting the conclusions that can be drawn. The end result is that, although a lot of very detailed work has been done, no real conclusion can be drawn from these experiments. The publication of this data therefore seems premature.

The rational for these experiments also does not appear entirely sound. Although the IF/IH motor neurons are labelled by the Atoh1cre the authors were unable to detect endogenous expression of Atoh1 in these cells, even using sensitive methods such as RNA-scope. This raises the possibility that the labelling is due to ectopic expression in the Atoh1cre mouse line. Any role for Atoh1 in the specification of IH and IF motor neurons remains unclear (and would require further investigation). The evidence supporting the idea that IH and IF motor neurons may send collaterals to the cerebellum because Atoh1 specifies many cerebellar projecting neurons therefore seems quite weak (since they do not provide any evidence for endogenous expression of Atoh1 in IH and IF motor neurons). The fact that recombination driven by Atoh1cre in IH/IF motor neurons may not reflect endogenous Atoh1 expression needs to be taken into consideration much more throughout the manuscript, and in particular when framing these experiments.

Given the link between Atoh1 and specification of IF/IH motor neurons is quite weak, including this work with the characterisation of the mouse line does not lead to a particularly cohesive story. Had the authors been able to successfully use the Atoh1cre line in these experiments then there would have been an obvious link, but unfortunately this was not the case.

5. The finding that viruses injected into the periphery can cross the blood brain barrier is interesting and probably worth reporting as a warning to others. However, it appears disconnected from the rest of the paper and a concern is that this finding will get missed in this paper as it is an incidental finding rather than a major component of the work.

6. Comments on specific figures/sets of data (some of these comments may apply to data that will not be included in a revised manuscript, but we thought it still useful to feedback our comments):

(i) A number of experiments (Fig. 5-7) are mainly descriptive and do not quantitate the data. Some of the experiments were performed only in 1-2 animals.

(ii) Figure 3: It should be clarified in the figure legend or results section text if the location of the IF MN pool is simply inferred from the dorsal position of TOM/HB9 co-expressing cells, or if CTB injections are used at each age to positively identify IF MN.

(iii) Figure 4: What percentage of IF MN are ERR3+ if the IF pool is labeled using CTB rather than relying on the TOM expression? This would provide additional data to support or refute the possibility that gamma-MNs are truly sparse in IF. Alternatively, a review of the literature for information on the relative abundance of muscle spindles in the IF and IH motor pools might provide additional insight.

(iv) Figure 6: Are TOM+ axons observed in ascending tracts at any spinal level? Labeled somata are clearly observed in 6B,C,D,E, but some indication/discussion of where IF motor axon collaterals go in the spinal cord would be helpful.
